# Management of suspected and known eosinophilic esophagitis—a nationwide survey in Austria

**DOI:** 10.1007/s00508-023-02198-0

**Published:** 2023-04-18

**Authors:** Philipp Schreiner, Lorenz Balcar, Hansjörg Schlager, Christian Madl, Alexander Ziachehabi, Markus Mader, Karin Steidl, Patrick Dinkhauser, Simon Reider, Werner Dolak, Clemens Dejaco, Hans Peter Gröchenig, Gottfried Novacek

**Affiliations:** 1 Division of Gastroenterology and Hepatology, Department of Internal Medicine III, Medical University of Vienna, Vienna, Austria; 2 Division of Gastroenterology and Hepatology, Department of Internal Medicine, Medical University of Graz, University Hospital Graz, Graz, Austria; 3 Division of Gastroenterology and Hepatology, Krankenanstalt Rudolfstiftung, Vienna, Austria; 4 Department of Internal Medicine II, Kepler Universitätsklinikum, Linz, Austria; 5 Department of Internal Medicine II, Universitätsklinikum St. Pölten, St. Pölten, Austria; 6 Department of Internal Medicine, Hospital Brothers of Mercy, St. Veit an der Glan, Austria; 7 Department of Internal Medicine I, Division of Gastroenterology and Hepatology, Endocrinology and Rheumatology, Klinikum Wels-Grieskirchen, Wels, Austria; 8 Department of Internal Medicine 2 (Gastroenterology and Hepatology), Faculty of Medicine, Kepler University Hospital, Johannes Kepler University, Linz, Austria

**Keywords:** Dysphagia, Esophageal Food Impaction, Esophageal Disease, Swallowing Disorder, Inflammatory Bowel Disease

## Abstract

**Introduction:**

Eosinophilic esophagitis (EoE) is a chronic immune-mediated disease of the esophagus with increasing incidence and dysphagia as the main symptom. The management of suspected or known EoE by Austrian endoscopists has not been investigated yet.

**Methods:**

A web-based survey with 13 questions about the management of EoE was sent to endoscopists via the Austrian Society of Gastroenterology and Hepatology (ÖGGH).

**Results:**

A total of 222 endoscopists (74% gastroenterologists, 23% surgeons, and 2% pediatricians; 68% working in a hospital) from all 9 states participated. In patients with dysphagia but a normal appearing esophagus, 85% of respondents reported always taking biopsies; however, surgeons were less likely to obtain biopsies compared to gastroenterologists (“always” 69% vs. 90%, “sometimes” 29% vs. 10%, “never” 2% vs. 0%, *p* < 0.001). The approved budesonide orodispersible tablet is the preferred first-line drug used in EoE, ahead of proton pump inhibitors (PPI). Only 65% of participants monitor the patients by endoscopy and histology after 12 weeks of induction therapy, 26% do not continue maintenance therapy, and 22% monitor patients only when symptomatic.

**Conclusion:**

The vast majority of Austrian endoscopists adhere to the European and US guidelines in cases of suspected EoE. In contrast, despite the chronic disease course, a significant percentage of providers indicate not to use maintenance therapy and monitor the patients routinely.

**Supplementary Information:**

The online version of this article (10.1007/s00508-023-02198-0) contains supplementary material, which is available to authorized users.

## Background

Eosinophilic esophagitis (EoE) is an immune-mediated disease of the esophagus with a rising incidence and a prevalence of 1–2 in 1000 people in western countries [1]. Nowadays, EoE represents the main cause for dysphagia and esophageal food impaction (EFI) in young adults. However, despite the growing knowledge and interest in EoE, the management of patients with dysphagia [2] and with an EFI [3] is often not in line with guidelines and varies widely between physicians. This is even true, although the American and European guidelines have the same recommendations [4, 5]. Furthermore, follow-ups are seldomly performed in patients with established diagnosis of EoE and rates of surveillance endoscopies with a regular biopsy protocol are low [6, 7].

Failure to take biopsies during bolus impaction or not obtaining biopsies in patients with dysphagia, may result in a long diagnostic delay [8]. In addition, inadequate monitoring and lack of maintenance therapy may lead to disease progression with fibrosis, repeated EFI and reduced quality of life.

A few years ago, a survey on the management of EoE demonstrated that adherence to guidelines is low among US gastroenterologists [6]. Additionally, a similar study conducted in Germany [2] confirmed the low adherence of gastroenterologists regarding the management in EoE. As interest in EoE has increased in recent years, it is hypothesized that nowadays physicians have a higher awareness than in the past and are more likely to adhere to guidelines.

However, current practice pattern of Austrian endoscopists in patients with suspected or known EoE is unknown. The aim of this study was therefore to analyze the management of suspected or known EoE among endoscopists in Austria and to compare the clinical practice with the current European and American recommendations [4, 5].

## Methods

We developed a web-based survey to analyze how endoscopists in Austria manage suspected or known EoE. The questionnaire comprised 8 questions of how dysphagia and EoE are managed and 5 sociodemographic questions (specialty, state of practice, practice setting, duration of practice, experience in the treatment of EoE patients). The survey was distributed by a link (surveymonkey) to all 1230 members of the Austrian Society of Gastroenterology and Hepatology (ÖGGH). Participation was possible between 3 and 30 December 2022. There was no incentive to participate in the survey and all responses were anonymous. Respondents provided consent at the beginning of the survey and had to be certified in endoscopy. Endoscopists in training were excluded from the survey. Next to descriptive statistics, we analyzed the difference in the management between surgeons and gastroenterologists/pediatricians and between hospital and private practice.

All statistical analyses were performed using IBM SPSS Statistics 27 (IBM, New York, NY, USA), or GraphPad Prism 8 (GraphPad Software, CA, USA). Categorical variables were reported as absolute (*n*) and relative frequencies (%). The χ^2^-test was used for group comparisons. Graphical depiction of results was chosen as appropriate. The level of significance was set at a 2-sided *p*-value < 0.05.

## Results

**Fig. 1:**
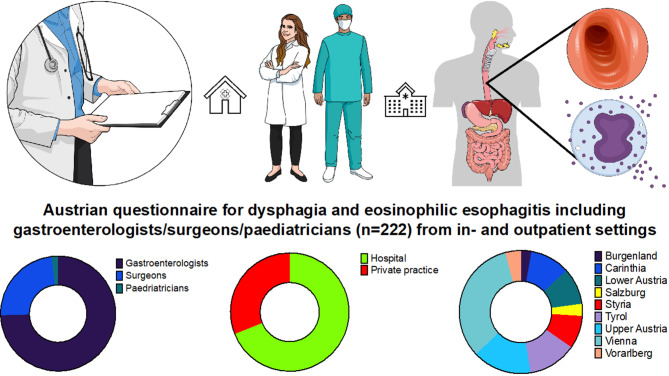
Study description

A total of 223 participants answered the questionnaire between 3 and 30 December 2022. After exclusion of one questionnaire that was completed by a provider not certified in endoscopy, a total of 222 surveys were analyzed. Respondents were classified as gastroenterologists (74%), surgeons (23%) or pediatricians (2%) (Fig. 1). Most (68%, 151) subjects predominantly work in a hospital-based setting, have experience in endoscopy more than 10 years (63%), but treat only 0–1 patients with EoE per month (76%). Endoscopists of every federal state participated in the questionnaire (Burgenland 3%, Carinthia 10%, Lower Austria 10%, Salzburg 3%, Styria 9%, Tyrol 13%, Upper Austria 15%, Vienna 32%, Vorarlberg 4%). All answers were compared with current guidelines and expert recommendations (Supplementary file 1).

### First diagnostic steps in patients with dysphagia

**Fig. 2:**
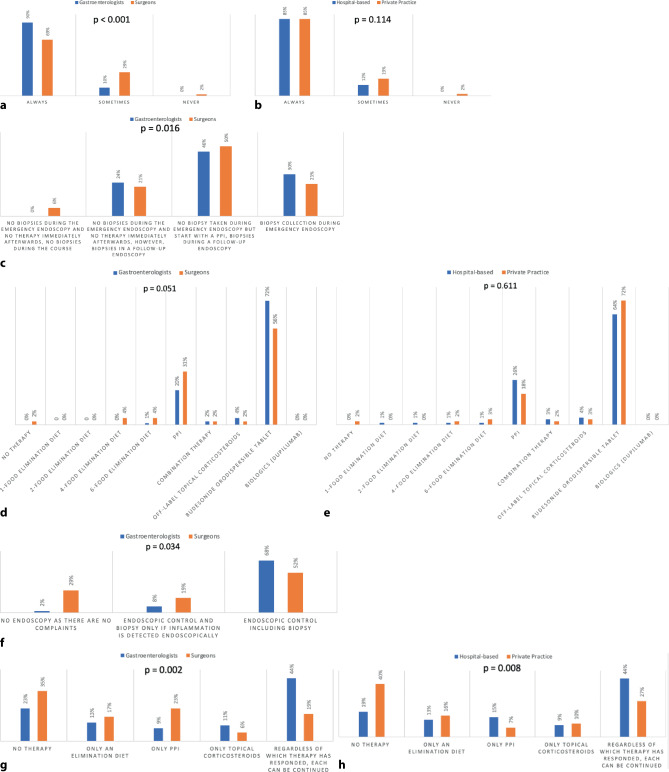
**a** Biopsy in patients with dysphagia with a normal esophagus: answers according to specialty **b** Biopsy in patients with dysphagia with a normal esophagus: answers according to practice setting. We analyzed the difference between groups (gastroenterology vs. surgery; hospital vs. private practice, resp.) using χ^2^-test. Respective *p*-values are shown in the figure. **c** Management in case of bolus impaction: answers according to specialty. We analyzed the difference between groups (gastroenterology vs. surgery) using χ^2^-test. Respective *p*-values are shown in the figure. **d** First-line drug in EoE: answers according to specialty and **e** First-line drug in EoE: answers according to practice setting. We analyzed the difference between groups (gastroenterology vs. surgery; hospital vs. private practice) using χ^2^-test. Respective *p*-values are shown in the figure. **f** Diagnostic procedure after 12 weeks of induction therapy: answers according to specialty. We analyzed the difference between groups (gastroenterology vs. surgery) using χ^2^-test. Respective *p*-values are shown in the figure. **g** Long-term management: Answers according to specialty **h** Long-term management: Answers according to practice setting. We analyzed the difference between groups (gastroenterology vs. surgery; hospital vs. private practice) using χ^2^-test. Respective *p*-values are shown in the figure

Most respondents perform an upper endoscopy as first step in patients with esophageal dysphagia (84%) and use to take biopsies routinely even in a normal appearing esophagus (85%). Although there is no difference between hospital-based vs. private practice respondents, surgeons obtain biopsies significantly less often than gastroenterologists (“always” 69% vs. 90%, “sometimes” 29% vs. 10%, “never” 2% vs. 0%, *p* < 0.001) (Fig. 2a, b). There is a heterogeneity among endoscopists in the numbers of obtained biopsies. Only half of subjects take > 4 biopsies in at least 2 sample containers (Table 1). In cases of an esophageal food impaction (EFI), most endoscopists do not take biopsies during the emergency endoscopy, but tend to start treatment with a proton pump inhibitor (PPI, 47%) and indicate to obtain biopsies at a later time point. However, gastroenterologists more commonly take biopsies during the emergency endoscopy compared to surgeons (30% vs. 23%, *p* = 0.016) (Fig. 2c).

**Table 1:** Answers of all respondents (*n* = 222) to the 8 questions of how dysphagia and EoE are managed

*Characteristics, n (%)*	***Study cohort*, *n*** **=** **222**
**What is your first measure in a patient with oesophageal dysphagia?**	
Esophagogastroduodenoscopy	186 (84%)
Empirical trial with a PPI	16 (7%)
Video kinematography	19 (9%)
Manometry	1 (0.5%)
**Do you take biopsies in a patient with dysphagia and an endoscopically unremarkable oesophagus?**	
Always	189 (85%)
Sometimes	32 (14%)
Never	1 (0.5%)
**If you suspect eosinophilic oesophagitis (EoE), how many biopsies and how many sample containers do you take?**	
1–2 biopsies into one sample container	1 (0.5%)
A total of 2 biopsies, but each biopsy in a different sample container	2 (1%)
A total of 3–4 biopsies in one sample containers	16 (7%)
A total of 3–4 biopsies in two different sample containers	29 (13%)
A total of 3–4 biopsies in three different sample containers	43 (19%)
> 4 biopsies into one sample container	21 (10%)
A total of > 4 biopsies in two different sample containers	46 (21%)
A total of > 4 biopsies in three different sample containers	64 (29%)
**In the case of endoscopic removal of a first-time bolus impaction (without a known diagnosis of EoE), what is your usual procedure?**	
No biopsies during the emergency endoscopy and no therapy immediately afterwards, no biopsies during the course	3 (1%)
No biopsies during the emergency endoscopy and no therapy immediately afterwards, however, biopsies in a follow-up endoscopy	52 (23%)
No biopsy taken during emergency endoscopy but start with a PPI, biopsies during a follow-up endoscopy	105 (47%)
Biopsy collection during emergency endoscopy	62 (28%)
**What is your preferred first-line therapy for diagnosed EoE?**	
No therapy	1 (0.5%)
1‑food elimination diet	1 (0.5%)
2‑food elimination diet	1 (0.5%)
4‑food elimination diet	2 (1%)
6‑food elimination diet	4 (2%)
PPI	52 (23%)
Combination therapy	5 (2%)
Off-label topical corticosteroids (fluticasone spray, budesonide sirup)	8 (4%)
(in adults) approved topical corticosteroid (Jorveza®)	148 (67%)
Biologics (dupilumab)	0 (0%)
**In case of clinical remission (free of symptoms) after 12 weeks of therapy, what is your usual diagnostic procedure?**	
No endoscopy as there are no complaints	54 (24%)
Endoscopic control and biopsy only if inflammation is detected endoscopically	24 (11%)
Endoscopic control including biopsy	144 (65%)
**Would you continue the initiated therapy (PPI, topical corticosteroids, or elimination diet) as a permanent therapy in patients in remission?**	
No therapy I would continue as maintenance therapy	57 (26%)
Only an elimination diet would I continue as maintenance therapy	31 (14%)
Only PPI I would continue as maintenance therapy	28 (13%)
Continue topical corticosteroids only as maintenance therapy	22 (10%)
Regardless of which therapy has responded (PPI, topical corticosteroids or elimination diet), continue each therapy as maintenance therapy (= continuation therapy)	84 (38%)
**Do you schedule regular check-ups for patients with an EoE?**	
No, only if the patient has complaints	48 (22%)
Yes, clinical control every 1–2 years	52 (23%)
Yes, clinical, endoscopic controls with biopsies every 1‑2 years	88 (40%)
Yes, every > 2 years clinical control	6 (3%)
Yes, every > 2 years clinical and endoscopic control with biopsies	28 (13%)

*PPI*

*Jorveza®*, Dr. Falk Pharma

### Management after diagnosis

The preferred first-line drug in a new diagnosis of EoE is an approved orodispersible budesonide tablet (67%) followed by PPI (23%). Although surgeons tended to less commonly state budesonide orodispersible tablets as first-line drug (56% vs. 72%, *p* = 0.051), there is no statistically significant difference in type of specialty or practice setting regarding choice of first-line treatment (Fig. 2d, e). In the case of clinical remission after 12 weeks, only 65% of endoscopists monitor the response by endoscopy and histology with significant differences between surgeons and gastroenterologists (Fig. 2f). A quarter of respondents (24%) do not perform an endoscopy in patients without symptoms after 12 weeks of induction therapy.

### Long-term management

Only 38% of all respondents would continue the initiated induction therapy as maintenance therapy. However, gastroenterologists and endoscopists working at a hospital setting continue maintenance treatment significantly more often (Fig. 2g, h). Although regular follow-ups with endoscopy and biopsies are performed by more than half (53%) of endoscopists, one fifth (22%) of subjects schedule follow-ups only in symptomatic patients.

## Discussion

This nationwide survey represents the first investigation of how suspected or known EoE is managed by Austrian endoscopists and demonstrates a substantial heterogeneity in management. The following main findings emerge: (1) most endoscopists, even though surgeons less often, obtain biopsies even in a normal appearing esophagus in patients with dysphagia; (2) the approved budesonide orodispersible tablet is the most preferred first-line medication in a new diagnosis of EoE; (3) there exists a hesitancy against a maintenance therapy and follow-up rates with endoscopy and histology are low.

Diagnostic delay in EoE has not changed in the last decades and is around 4 years [8]. As patient-dependent delay is difficult to address, it is even more important that the physician-dependent delay is as short as possible. An endoscopist encounters a patient with an unknown EoE either at the emergency department with an EFI or in the case of an upper endoscopy due to esophageal symptoms. Therefore, it is of utmost importance that all patients with esophageal symptoms, especially dysphagia, and patients with an EFI should undergo biopsies following an adequate protocol (at least 6 biopsies) regardless of the endoscopic appearance of the esophagus [4, 9]. In comparison to a survey conducted in Germany some years ago, a higher rate of respondents in Austria (85%) obtains biopsies in patients with dysphagia and an endoscopically normal appearing esophagus. However, although guidelines recommend taking at least 5–6 biopsies of different locations [4], only 50% of endoscopists indicate to obtain more than 4 biopsies. On the one hand, it is affirmative to see that there is increased awareness regarding EoE resulting in a high number of patients that will be biopsied, but on the other hand it would be desirable if sufficient biopsies were taken in these patients in order not to miss EoE due to the patchy character of the disease.

Similar to a survey conducted in Europe and the USA [3], many non-EoE experts are unaware of the importance of taking biopsies at the time of esophageal food impaction (EFI). Nearly 50% of endoscopists in Austria indicate not to take biopsies during the emergency endoscopy in case of EFI, but to start a PPI and postpone the biopsies to a follow-up endoscopy. Although this approach may be justified because of real-life obstacles (patient often not fasting and no endoscopy nurse available), it goes along with problems that should be avoided. Patients without having had biopsies at the index endoscopy will be lost to follow-up in up to 80% of cases [10] and most of these patients are likely to be left with an undiagnosed EoE [11]. Furthermore, if PPI are not stopped before the follow-up endoscopy, it will mask EoE [12] resulting in patients with a missed diagnosis.

Nowadays, a budesonide orodispersible tablet is the only approved drug for the treatment of EoE in Europe. Nevertheless, PPI are by far the most prescribed drug in patients with EoE in many countries in Europe [13]. It is hypothesized that the orodispersible tablet is favored by the large majority as first-line therapy in Austria due to the fact that the data for induction therapy are very convincing [14, 15] and reimbursement, at least for the first months, is covered by the health insurance in Austria.

After 12 weeks of induction therapy, guidelines recommend an endoscopy with biopsies to assess mucosal healing or at least histologic response [4]. Due to the discordance between symptoms and inflammation, a symptom-based follow-up is not appropriate [16]. However, in contrast to Germany [2], where 84.6% of gastroenterologists monitor patients with endoscopy and histology after initiation of therapy, only 65% of respondents in Austria follow these guidelines.

As EoE is a chronic disease with a progressive disease course in the majority of patients and has a 90% relapse rate after cessation of therapy [17], a long-term treatment is highly important and follow up is needed on a regular basis. Not in line with recommendations, barely 40% percent of responders would continue the initiated therapy as maintenance therapy. Interestingly, there was a significant difference between gastroenterologists and surgeons (44% vs. 19%) pointing out that surgeons have a greater reluctance to prescribe a maintenance treatment. The reason of this hesitancy against a long-term therapy in a known chronic inflammatory gastrointestinal disease is ambiguous. Whether a lack of knowledge of the chronic nature of disease, the scepticism towards Swallowed topical corticosteroids (STC) or missed insurance coverage for maintenance treatment of the approved medication in EoE is responsible for this finding, cannot be answered. It must be emphasized that in patients treated with the approved orodispersible tablet there is no safety signal and sustained efficacy up to 96 weeks of maintenance treatment [18]. Furthermore, real-life data with other STC (mostly fluticasone) from Switzerland confirms efficacy and safety over many years [19].

Although scientific data demonstrate the importance of a follow-up every 12–24 months [20, 21], the literature regarding the adequate time interval for performing EGD in patients with stable disease is unknown. Although a regular and close follow-up (at least every 2 years) is advocated by most experts [9, 22], only half of respondents indicate to schedule regular check every 1–2 years.

Our study has several strengths and also some limitations. This is the first survey to investigate the clinical practice pattern of Austrian endoscopists regarding suspected and known EoE. We were not able to determine the exact response rate because the survey was not sent directly to the participants but distributed by the Austrian Society of Gastroenterology and Hepatology (ÖGGH) and members were allowed to forward the link to participate. The ÖGGH has 1230 members including not only endoscopists, but also physicians not certified in endoscopy, retired physicians and physicians in training. The survey was sent out to all 1230 ÖGGH members originally. As only certified endoscopists were allowed to participate in the survey, our response rate may be similar to the survey conducted in Germany with 413 respondents [2].

In conclusion, our survey demonstrates that endoscopists in Austria have a high awareness of EoE in patients with dysphagia, but there is a wide heterogeneity of clinical practice pattern in terms of biopsy protocol, long-term treatment and follow-up management in patients with EoE. Our results may help to indicate where there is room for improvement to ameliorate adherence to the guidelines and ultimately improve the management of patients with EoE.

## Supplementary Information

Guidelines and expert recommendations

## References

[1] de Rooij WE, Barendsen ME, Warners MJ, van Rhijn BD, Verheij J, Bruggink AH (2021). Emerging incidence trends of eosinophilic esophagitis over 25 years: results of a nationwide register-based pathology cohort. Neurogastroenterol Motil.

[2] Miehlke S, von Arnim U, Schlag C, Frieling T, Madisch A, Loibl R (2019). Clinical management of eosinophilic esophagitis—a nationwide survey among gastroenterologists in Germany. Z Gastroenterol.

[3] Schreiner P, Safroneeva E, Schoepfer A, Greuter T, Biedermann L, Schlag C (2022). Management of eosinophilic esophagitis associated food impaction in Europe and the United States. Dis Esophagus.

[4] Lucendo AJ, Molina-Infante J, Arias A, von Arnim U, Bredenoord AJ, Bussmann C (2017). Guidelines on eosinophilic esophagitis: evidence-based statements and recommendations for diagnosis and management in children and adults. United European Gastroenterol J.

[5] Hirano I, Chan ES, Rank MA, Sharaf RN, Stollman NH, Stukus DR (2020). AGA institute and the joint task force on allergy-immunology practice parameters clinical guidelines for the management of eosinophilic esophagitis. Gastroenterology.

[6] Chang JW, Saini SD, Mellinger JL, Chen JW, Zikmund-Fisher BJ, Rubenstein JH (2019). Management of eosinophilic esophagitis is often discordant with guidelines and not patient-centered: results of a survey of gastroenterologists. Dis Esophagus.

[7] Zifman E, Banai H, Shamir R, Ringel-Kulka T, Zevit N (2018). Practice differences in the diagnosis and management of eosinophilic esophagitis among adult and pediatric gastroenterologists in Israel. J Pediatr Gastroenterol Nutr.

[8] Murray FR, Kreienbuehl AS, Greuter T, Nennstiel S, Safroneeva E, Saner C (2022). Diagnostic delay in patients with eosinophilic esophagitis has not changed since the first description 30 years ago: diagnostic delay in eosinophilic esophagitis. Am J Gastroenterol.

[9] Dhar A, Haboubi HN, Attwood SE, Auth MKH, Dunn JM, Sweis R (2022). British society of gastroenterology (BSG) and British society of paediatric gastroenterology, hepatology and nutrition (BSPGHAN) joint consensus guidelines on the diagnosis and management of eosinophilic oesophagitis in children and adults. Gut.

[10] Chang JW, Olson S, Kim JY, Dolan R, Greenson J, Sanders G (2019). Loss to follow-up after food impaction among patients with and without eosinophilic esophagitis. Dis Esophagus.

[11] Murray FR, Kreienbuhl A, Straumann A, Biedermann L, Schreiner P (2022). Natural history of patients lost to follow-up after esophageal food impaction. Clin Gastroenterol Hepatol.

[12] Hillman L, Donohue S, Broman AT, Hoversten P, Gaumnitz E, Lomeli L (2021). Empiric proton pump inhibitor therapy after esophageal food impaction may mask eosinophilic esophagitis diagnosis at follow-up. Dis Esophagus.

[13] Laserna-Mendieta EJ, Casabona S, Savarino E, Perello A, Perez-Martinez I, Guagnozzi D (2020). Efficacy of therapy for eosinophilic esophagitis in real-world practice. Clin Gastroenterol Hepatol.

[14] Lucendo AJ, Miehlke S, Schlag C, Vieth M, von Arnim U, Molina-Infante J (2019). Efficacy of budesonide orodispersible tablets as induction therapy for eosinophilic esophagitis in a randomized placebo-controlled trial. Gastroenterology.

[15] Straumann A, Lucendo AJ, Miehlke S, Vieth M, Schlag C, Biedermann L (2020). Budesonide orodispersible tablets maintain remission in a randomized, placebo-controlled trial of patients with eosinophilic esophagitis. Gastroenterology.

[16] Safroneeva E, Straumann A, Coslovsky M, Zwahlen M, Kuehni CE, Panczak R (2016). Symptoms have modest accuracy in detecting endoscopic and histologic remission in adults with eosinophilic esophagitis. Gastroenterology.

[17] Greuter T, Bussmann C, Safroneeva E, Schoepfer AM, Biedermann L, Vavricka SR (2017). Long-term treatment of eosinophilic esophagitis with swallowed topical corticosteroids: development and evaluation of a therapeutic concept. Am J Gastroenterol.

[18] Biederman L (2022). Budesonide orodispersible tablets are able to maintain clinical, histological and endoscopic remission in adult patients with eosinophilic esophagitis: results from the 96-weeks open-label extension phase following the 1-year double-blind EOS-2 trial.

[19] Greuter T, Godat A, Ringel A, Almonte HS, Schupack D, Mendoza G (2021). Effectiveness and safety of high—vs low-dose swallowed topical steroids for maintenance treatment of eosinophilic esophagitis: a multicenter observational study. Clin Gastroenterol Hepatol.

[20] Bon L, Safroneeva E, Bussmann C, Biedermann L, Schreiner P, Vavricka SR (2022). Close follow-up is associated with fewer stricture formation and results in earlier detection of histological relapse in the long-term management of eosinophilic esophagitis. United European Gastroenterol J.

[21] Chang NC, Thakkar KP, Ketchem CJ, Eluri S, Reed CC, Dellon ES (2022). A gap in care leads to progression of fibrosis in eosinophilic esophagitis patients. Clin Gastroenterol Hepatol.

[22] Leiman DA, Kamal AN, Otaki F, Bredenoord AJ, Dellon ES, Falk GW (2023). Quality indicators for the diagnosis and management of eosinophilic esophagitis. Am J Gastroenterol.

