# Supplementary material for: Management of suspected and known eosinophilic esophagitis—a nationwide survey in Austria
Source: Wien Klin Wochenschr. 2023 Apr 18;135(15-16):406–13. doi: 10.1007/s00508-023-02198-0 (PMC10444684; doi:10.1007/s00508-023-02198-0)
Supplement: Supplementary file 1 — Guidelines and expert recommendations [file 508_2023_2198_MOESM1_ESM.docx]

**Supplementary File 1: Guidelines^1-3^ and expert recommendations^4^**

**What is your first measure in a patient with oesophageal dysphagia?**

*A recommended algorithm in patients with dysphagia does NOT exist. Even though the quality of evidence is low, most experts recommend as a first step a upper endoscopy with/without barium esophagogram/videokinematography*

**Do you take biopsies in a patient with dysphagia and an endoscopically unremarkable oesophagus?**

*Biopsies should be taken in all patients with dysphagia despite a normal endoscopic appearance of the esophagus*

**If you suspect eosinophilic oesophagitis (EoE), how many biopsies and how many sample containers do you take?**

*At least 6 biopsies from at least two different locations of the esophagus should be obtained*

**In the case of endoscopic removal of a first-time bolus impaction (without a known diagnosis of EoE), what is your usual procedure?**

*Expert opinion and the British society of Gastroenterology guidelines from 2022 recommend to take biopsies at the index endoscopy in patients presenting with food bolus obstruction*

**What is your preferred first-line drug therapy for diagnosed EoE?**

*Proton-pump inhibitors (PPI), swallowed topical corticosteroids (STC) or diet (elemental or empiric elimination diet) can be used as first line therapies in patients with EoE. However, the AGA only recommends STC with a STRONG recommendation*

**In case of clinical remission (free of symptoms) after 12 weeks of therapy, what is your usual diagnostic procedure?**

*Since symptoms correlate only modest with histologic activity, an endoscopy with biopsies is recommended to assess response after 8-12 weeks of initiated therapy*

**Would you continue the initiated therapy (PPI, topical corticosteroids, or elimination diet) as a permanent therapy in patients in remission?**

Since EoE is a chronic inflammatory disease with a high relapse rate after withdrawal of therapy, a maintenance therapy should be continued

***Do you schedule regular check-ups for patients with an EoE?***

*After remission is achieved, regular clinical and endoscopic follow-up is recommended (expert opinion only)*

*References*

1. Lucendo AJ, Molina-Infante J, Arias A, et al. Guidelines on eosinophilic esophagitis: evidence-based statements and recommendations for diagnosis and management in children and adults. *United European Gastroenterol J.* 2017;5(3):335-358.

2. Hirano I, Chan ES, Rank MA, et al. AGA Institute and the Joint Task Force on Allergy-Immunology Practice Parameters Clinical Guidelines for the Management of Eosinophilic Esophagitis. *Gastroenterology.* 2020;158(6):1776-1786.

3. Dhar A, Haboubi HN, Attwood SE, et al. British Society of Gastroenterology (BSG) and British Society of Paediatric Gastroenterology, Hepatology and Nutrition (BSPGHAN) joint consensus guidelines on the diagnosis and management of eosinophilic oesophagitis in children and adults. *Gut.* 2022;71(8):1459-1487.

4. Leiman DA, Kamal AN, Otaki F, et al. Quality Indicators for the Diagnosis and Management of Eosinophilic Esophagitis. *Am J Gastroenterol.* 2023.
